# The Impact of a Digital Peer-Supported App on Daily Steps and Lifestyle Changes Among Individuals With Prediabetes and Early-Stage Type 2 Diabetes: Prospective, Nonrandomized Controlled Trial

**DOI:** 10.2196/75953

**Published:** 2025-12-15

**Authors:** Shota Yoshihara, Takeo Shibata, Mizuki Kosone, Megumi Shibuya, Kayoko Takahashi

**Affiliations:** 1 Department of Rehabilitation Sciences Graduate School of Medical Sciences Kitasato University Kanagawa Japan; 2 Department of Business Development A10 Lab Inc Tokyo Japan; 3 Department of Health Management School of Health Studies Tokai University Kanagawa Japan; 4 Department of Occupational Therapy School of Allied Health Science Kitasato University Kanagawa Japan

**Keywords:** apps, digital peer support app, eHealth, mHealth, mobile health, mobile phone, peer support, type 2 diabetes mellitus, prediabetes

## Abstract

**Background:**

Physical activity is a simple, low-risk intervention that could be integrated into daily life to improve glycemic control in individuals with prediabetes and early-stage type 2 diabetes mellitus (T2DM). However, maintaining physical activity remains challenging, even when its benefits are well understood. Although digital peer support has the potential to promote and maintain physical activity, its effectiveness has not yet been sufficiently established.

**Objective:**

This study examined the impact of a digital peer-supported app on daily step goal achievement and average daily step counts among individuals with prediabetes and early-stage T2DM.

**Methods:**

This 3-month, prospective, nonrandomized controlled trial recruited participants aged 40-79 years with prediabetes or early-stage T2DM. The participants were divided into a digital peer-supported app group and a control group. The digital peer-supported app group tracked their daily steps, shared their progress with small peer groups, and received real-time feedback and support within the app. The control group tracked their steps individually using a pedometer. The primary outcome was the achievement rate of daily step goals. Secondary outcomes included the average daily step count, BMI, glycosylated hemoglobin A_1c_ level, blood pressure, and self-reported lifestyle behaviors.

**Results:**

A total of 32 participants (digital peer-supported app group: n=18 and control group: n=14) completed the study. The digital peer-supported app group reported a significantly higher median daily step goal achievement rate (57.2%, IQR 32.2%-90% vs 26.7%, IQR 10%-64.4%; *P*=.04) and daily step count (6854, IQR 4846-10388 steps vs 3946, IQR 3176-6832 steps; *P*=.03) compared to the control group. No significant differences were observed in glycosylated hemoglobin A_1c_ levels, blood pressure, BMI, or lifestyle behaviors.

**Conclusions:**

Our findings inform research in this field by suggesting that a digital peer-supported app may support daily step goal achievement and increase step counts among individuals with prediabetes and early-stage T2DM over the 3-month study period. The digital peer-supported app facilitated real-time feedback, peer approval, and continuous engagement to support participation in light physical activity.

**Trial Registration:**

UMIN-CTR UMIN000039466; https://center6.umin.ac.jp/cgi-open-bin/ctr_e/ctr_view.cgi?recptno=R000044999

## Introduction

Type 2 diabetes mellitus (T2DM) is a global public health issue that increases health care costs, strains health care systems, and reduces life expectancy and quality of life [1]. Unhealthy lifestyle behaviors, such as excessive consumption of fat, sugar, and alcohol, as well as insufficient physical activity, contribute significantly to the development of T2DM [2].

Previous studies have shown that physical activity is an important nonpharmacological intervention for the management of patients with T2DM [3-5]. Among the various forms of physical activity, walking is a beneficial form of physical activity that can be easily integrated into daily commuting for individuals with T2DM, including busy workers and older adults, with minimal risk of adverse effects [6]. A meta-analysis of 20 randomized controlled trials (RCTs) reported that walking significantly decreased glycosylated hemoglobin A_1c_ (HbA_1c_) by 0.50% (95% CI –0.78% to –0.21%) among patients with T2DM [7]. Similarly, another meta-analysis including 10 RCTs showed that individuals with prediabetes showed a decrease in HbA_1c_ levels after engaging in aerobic training compared to the control group, with a weighted mean difference of 0.34% (95% CI –0.45 to –0.23) [8].

Individuals with prediabetes and T2DM often struggle to enhance and maintain their physical activity, including walking [9]. However, several previous studies have reported that peer support can be an effective form of external assistance in addressing this challenge [10-12]. Nevertheless, traditional lifestyle counseling in clinical settings, such as peer support, requires significant human and time resources and is often provided through face-to-face interactions [13].

To address the limitations of conventional peer support, previous studies have examined digital peer support, which involves group-based apps that enable users to encourage each other toward healthy behaviors [14-16]. Given the evidence from face-to-face peer support studies showing increased physical activity, including walking, digital peer support may also be a promising approach for promoting physical activity, as measured by step counts. Moreover, amid growing interest in diverse digital tracking technologies, including continuous glucose monitoring [17], smartphone-derived step counts offer low-burden, automatically captured, dynamic data that are scalable across populations and may complement contemporary diabetes care.

Therefore, in this study, we aimed to examine the effect of a digital peer support app on the achievement rate of daily step goals and average daily step count, recorded by smartphones, among individuals with prediabetes and early-stage T2DM compared to nondigital peer support. If implemented effectively, the digital peer-supported app can facilitate step count increases in a convenient, cost-effective, and remote manner.

## Methods

### Study Settings

This study was a 3-month, prospective, non-RCT conducted between October and December 2019 as part of the Kanagawa “ME-BYO Living Lab” program [18]. The program partners with local municipalities, health-conscious companies (in this study, A10 Lab Inc, the developer and operator of the digital peer-supported app), and academic institutions that conducted independent evaluations of program effectiveness, providing a real-world evaluation platform for assessing the functionality and effectiveness of health-related interventions and their public health impact. Within this framework, A10 Lab Inc submitted the proposal and supplied the intervention app; program-operating funds supported this study. The protocol was reviewed by the Ethics Review Committee of Healthcare Systems Co, Ltd, a private company ethics committee retained under the program. Local municipalities assisted with participant recruitment at the request of A10 Lab Inc.

### Participants

Participants were recruited from individuals living or working in Kanagawa, Japan, through a municipality-led program aimed at increasing step counts for diabetes management. Participant recruitment was conducted through the dissemination of study invitations via posters, leaflets, and municipal public relations newsletters. Interested individuals were required to complete a preliminary registration via email.

Eligible participants were adults aged 40-79 years with prediabetes and early-stage T2DM, defined as HbA_1c_ levels between 5.6% and 7.0%. Participants were required to own a mobile phone. Individuals with severe diabetes-related symptoms or disabilities, those diagnosed with conditions that could interfere with the study, and those deemed unsuitable by the research team were excluded. The HbA_1c_ range of 5.6%-7.0% was selected to align with the municipal referral program, identifying prediabetes (5.6%-6.4%) and early-stage T2DM (≥6.5%), and to reflect the real-world behavioral focus of the intervention on increasing step counts via peer support.

Participants self-selected into either the digital peer-supported app group or the control group (with no digital peer-supported app). Although initially planned, randomization was not undertaken as the study was embedded in a municipality-led public program that prioritized participant autonomy. Eligibility was determined from recent health checkups or routine clinical testing results, and participants continued their usual clinical care throughout the trial. As the intervention comprised a minimal-risk behavioral program (step-count promotion with peer support) that did not alter medical management, no pre-enrollment medical examination or physician clearance was required, and no formal physician-verification workflow was implemented. Four participants who owned only feature phones (nonsmartphones) were placed in the control group and were provided with pedometers because they were unable to use the app. In addition, 3 participants who owned smartphones but lacked the technical skills to use the app were assigned to the control group for feasibility reasons.

Participants continued usual care throughout the trial. Medication use and related modifications, including those concerning glucose-lowering agents (eg, metformin, GLP-1 receptor agonists, SGLT2 inhibitors, and insulin), antihypertensives, or lipid-lowering therapies, were not systematically recorded, and no restrictions were imposed on routine prescribing.

### Program

All participants attended a face-to-face program introduction and set personal daily step goals for prediabetes and early-stage T2DM prevention and management. They were then divided into a digital peer-supported app group and a control group. The digital peer-supported app group received a tutorial during their initial login and used the app for 3 months. They could track their daily steps and work toward their goals as part of a team of up to 5 members, posting their step count and a photo once a day to share their progress and encourage mutual support. In contrast, the control group tracked their steps using a smartphone pedometer app, but worked individually without team participation, posting, or progress sharing.

Participants completed questionnaires and health checks (eg, sex, age, height, weight, HbA_1c_ level, and blood pressure) at the beginning and end of the program to assess changes in lifestyle habits and health behaviors. All participants submitted their 24-hour step counts via smartphones using a dedicated QR code at the end of the study.

### Intervention of Digital Peer-Supported Apps

**Figure 1:**
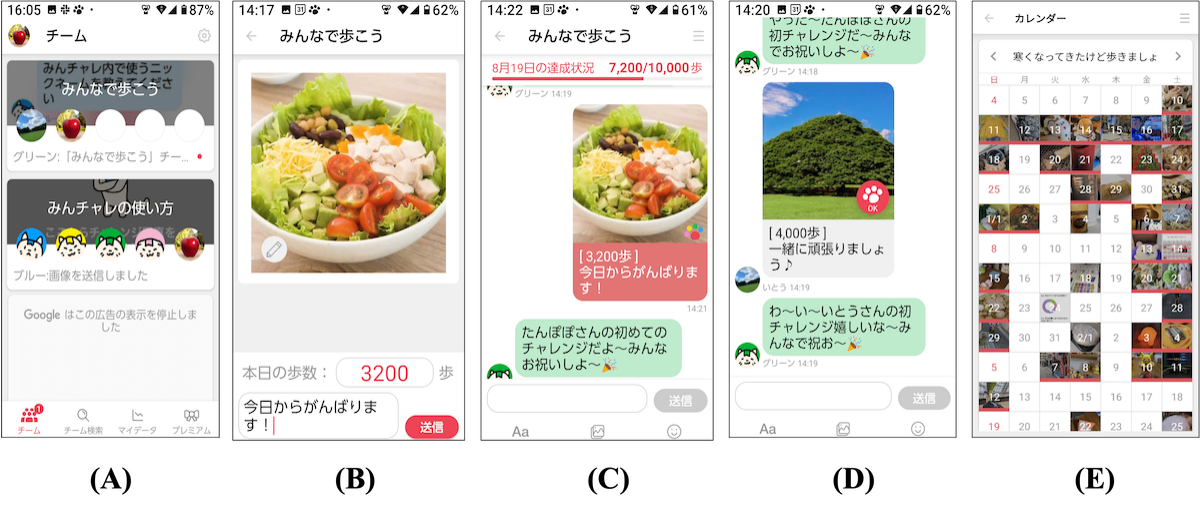
Examples of app screens. (A) Select a group, (B) post photos, view step counts, and comment within the group, posting a picture taken that day and commenting on the day’s events; (C) view the contents of the posts in the group, (D) respond to the posts by group members, and (E) check the records.

In this study, we used Minchalle (A10 Lab Inc), a commercially available digital peer-supported app for both Android and iPhone [14-16]. Originally developed to help users build desirable habits such as exercising, dieting, and learning English, the app has been downloaded over 1.5 million times. For this study, the app was customized to promote daily step achievement through anonymous group chats with up to 5 participants (Figure 1). Participants in the digital peer-supported app group posted their step counts, photos, and comments on the group chat once a day.

**Figure 2:**
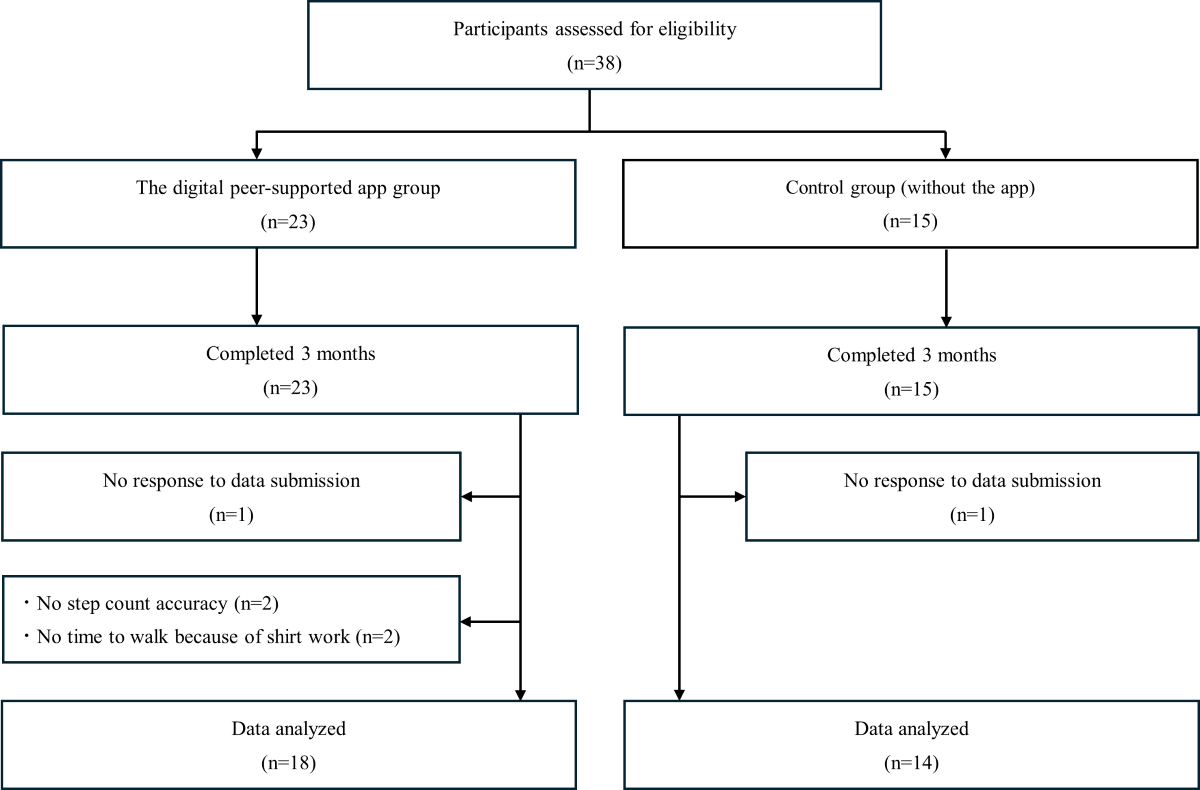
Flowchart of the study participants.

**Figure 3:**
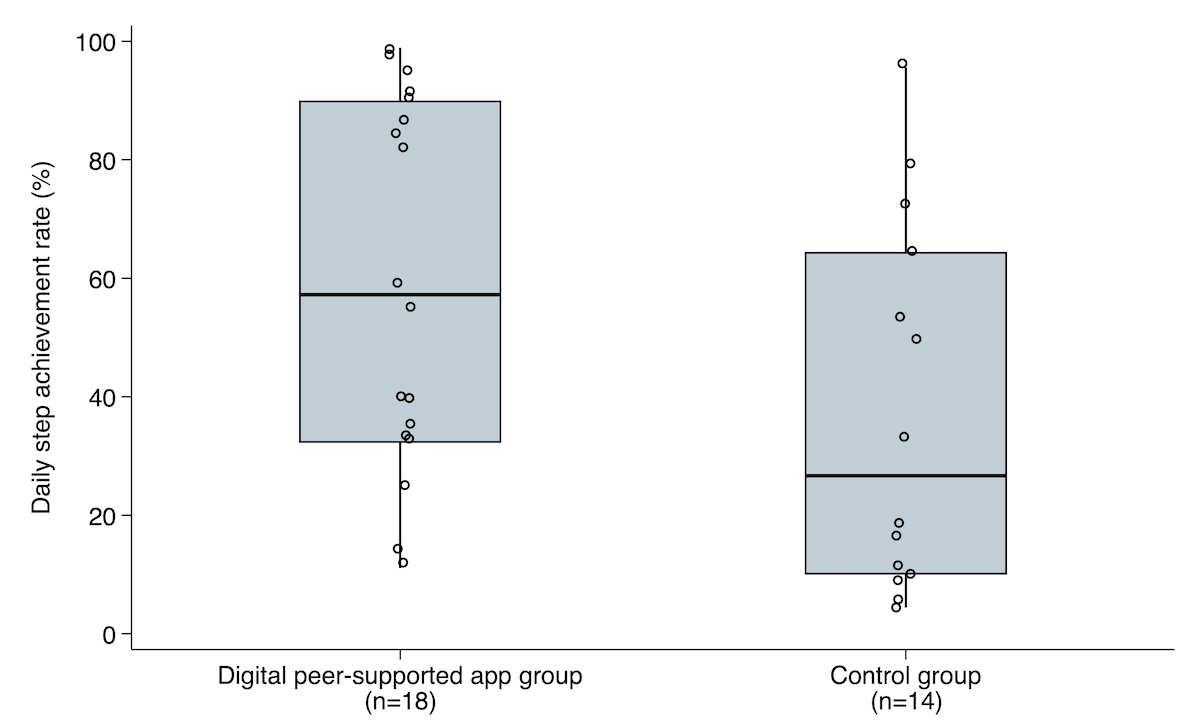
Comparison of daily step achievement rates between the digital peer-supported app and control groups. The median daily step achievement rate was significantly higher in the digital peer-supported app group (57.2%, IQR 32.2%-90%) than in the control group (26.7%, IQR 10%-64.4%), as examined using the Mann-Whitney U test (*P*=.04).

The main functions of the app were as follows: (1) posting step counts, photos, and comments about the day (Figures 1A-C); (2) receiving approval from group members (Figure 1D); (3) setting step-count goals at the group level; (4) receiving feedback on the team’s total daily step count; and (5) automatic group removal if a participant remained inactive for 15 days. Step counts were recorded using a smartphone, either manually or automatically, via Google Fit or Apple Health, and the app reported the daily step count at the time of posting. Participants could post comments or photos multiple times a day and interact with other members; however, this was not mandatory. The app was available to participants at no cost (Multimedia Appendix 1).

### Measurements

Participants’ demographic characteristics were assessed using a questionnaire that included sex (male or female), age group (40-49, 50-59, 60-69, or 70-79 years), number of cohabiting family members (continuous), occupational status (full-time or regular employee, self-employed, part-time or temporary worker, homemaker, or unemployed), and diabetes care support (yes or no). In addition, participants reported their average daily step count over the past month (<1000, 1000-1999, 2000-2999, 3000-3999, 4000-4999, 5000-5999, 6000-6999, 7000-7999, 8000-8999, 9000-9999, ≥10000, or unknown). They were also asked to specify their daily step goals (steps/day) to assess individual physical activity targets (continued).

Our primary endpoint was the achievement rate of average daily step goals over the 3-month period. The achievement rate was calculated as the number of days in which the participants exceeded their individually set step goals, recorded via smartphones and pedometers, divided by the total number of study days. The secondary endpoint was the average daily step count during the study period. Additional secondary outcomes included BMI, HbA_1c_ levels, blood pressure, and lifestyle questionnaire responses. BMI was calculated by dividing weight (kg) by height (m^2^). Participants completed a web-based questionnaire and had their weight, blood pressure, and HbA_1c_ levels measured at clinics or other facilities at baseline and after 3 months.

The same assessment schedule was applied for the lifestyle questionnaire, which evaluated the health status, lifestyle behaviors, and physical activity. Participants rated their overall health status (good, fairly good, not very good, or poor). Balanced meal consumption (staple food, main dish, and side dishes at least twice per day) was reported as (almost every day, 4-5 days per week, 2-3 days per week, or rarely). Healthy eating habits were assessed based on the frequency with which participants consciously selected healthy foods and practiced mindful eating (always, often, rarely, or never). Engagement in moderate-intensity physical activity (≥30 minutes/session) was categorized based on frequency as 2 or more times per week, once per week, 2-3 times per month, once per month, or rarely. Confidence in maintaining regular physical activity was rated on a 4-point scale (very confident, confident, not very confident, and not confident at all). Participants also reported their average daily sleep duration over the past month (<5, 5-6, 6-7, 7-8, 8-9, or ≥9 hours). Alcohol consumption frequency was recorded as daily, 3-4 times per week, 1-2 times per week, 1-2 times per month, or rarely or never. Finally, perceived stress levels were assessed on a 4-point scale as very high, high, low, or very low.

### Statistical Analysis

The Pearson chi-square test was used to confirm the categorical variable homogeneity between the 2 groups at baseline, and the Mann-Whitney *U* test was used for continuous variables.

The primary outcome was analyzed using the Mann-Whitney *U* test to compare daily step achievement rates between the digital peer-supported and control groups. Secondary outcomes included the daily average step count, analyzed using the Mann-Whitney *U* test. Changes in BMI, HbA_1c_ levels, and blood pressure were assessed using within-group *P* values calculated using the Wilcoxon signed-rank test and between-group *P* values calculated using the Mann-Whitney *U* test. Questionnaire-based analyses were conducted to examine lifestyle behavior–related changes. The Stuart-Maxwell test was used to evaluate within-group changes in categorical variable proportions over 3 months, and between-group comparisons were again performed using the Mann-Whitney *U* test.

To test the robustness of results, we performed a linear mixed-effects model with fixed effects for groups, days, and their interactions, and a random intercept for each participant to examine group-level trends in daily average step counts over time. Moreover, separate mixed-effects models were fitted within each group to assess within-group trends. Days with 0 recorded steps were treated as missing, assuming nonwear or device error, and were imputed using each participant’s average daily step count derived from their nonmissing data.

All analyses were performed using the Stata statistical software (version 18.0; StataCorp LLC) and IBM SPSS Statistics (version 24.0; IBM Corp). Two-tailed *P* values <.05 were considered statistically significant.

### Ethical Considerations

All participants provided written informed consent before their enrollment. This study adhered to the ethical principles outlined in the Declaration of Helsinki and was approved by the independent Ethics Review Committee of Healthcare Systems Co, Ltd, a private company ethics committee (approval no 1911). All collected data were anonymized before analysis to ensure participant confidentiality and privacy.

All participants who completed the study received a JPY 5000 (approximately US $46.26) gift card as compensation, irrespective of whether they submitted end-of-study self-reported outcomes. The 3-month endline survey invitation and compensation distribution were issued concurrently. The trial was registered with the University Hospital Medical Information Network (UMIN) Clinical Trials Registry (UMIN000039466).

## Results

Thirty-eight participants were initially enrolled in this study. In the digital peer-supported app group, 1 participant did not respond at the time of follow-up, 2 participants were excluded because of inaccurate step count data, and 2 participants were excluded because they had no time to walk due to shift work. One participant in the control group who did not respond at the time of the follow-up was excluded. The final statistical analysis included 18 participants in the digital peer support app group and 14 in the control group. Figure 2 shows a flowchart of the study participants.

Table 1 shows the baseline characteristics of the participants in the digital peer-supported app (n=18) and control (n=14) groups. Age, sex, household composition, and employment status were similar between the 2 groups. No notable differences were observed in the prevalence of diabetes care support. Moreover, physical measurements (BMI, HbA_1c_ level, and blood pressure) were comparable between the groups, and there were no significant differences between the groups in terms of average daily step counts and daily step goals. The proportion with prediabetes (HbA_1c_ 5.6%-6.4 %) was 15/18 (83.3%) and 11/14 (78.6%) in the digital peer-supported app and control groups, respectively, with no statistically significant differences. Therefore, baseline characteristics were considered well-balanced between the 2 groups.

**Table 1:** Baseline participant characteristics.

Variable			Digital peer-supported app group (n=18)		Control group (n=14)		*P* value	
**Age (years), n (%)**								.98
	40-49	2 (11.1)		2 (14.3)				
	50-59	7 (38.9)		5 (35.7)				
	60-69	7 (38.9)		5 (35.7)				
	70-79	2 (11.1)		2 (14.3)				
**Sex, n (%)**								.75
	Male	8 (44.4)		7 (50)				
	Female	10 (55.6)		7 (50)				
**Number of household members, n (%)**								.33
	Living alone	2 (11.1)		2 (14.3)				
	2 people	10 (55.6)		5 (35.7)				
	3 people	2 (11.1)		4 (28.6)				
	4 people	4 (22.2)		1 (7.1)				
	5 people	0 (0.0)		1 (7.1)				
	7 people	0 (0.0)		1 (7.1)				
**Employment status, n (%)**								.33
	Full-time employee	9 (50)		5 (35.7)				
	Self-employed	1 (5.6)		2 (14.3)				
	Part-time worker	1 (5.6)		3 (21.4)				
	Homemaker	0 (0.0)		1 (7.1)				
	Unemployed	7 (38.9)		3 (21.4)				
Support for diabetes care, n (%)			13 (72.2)		7 (50)		.20	
BMI (kg/m²), median (IQR)			24 (21-26)		23 (22-26)		.88	
HbA_1c_^a^ (%), median (IQR)			6.0 (5.6-6.4)		6.0 (5.7-6.4)		.74	
Prediabetes (HbA_1c_ 5.6%-6.4 %), n (%)			15 (83.3)		11 (78.6)		.73^b^	
Early-stage T2DM^c^ (HbA_1c_ 6.5%-7%), n (%)			3 (16.7)		3 (21.4)		—^d^	
Systolic blood pressure (mm Hg), median (IQR)			120 (110-128)		126 (121-134)		.22	
Diastolic blood pressure (mm Hg), median (IQR)			77 (70-81)		76 (72-79)		.97	
**Average daily step count during the past month, n (%)**								.26
	<1000	1 (5.6)		0 (0.0)				
	1000-<2000	2 (11.1)		5 (35.7)				
	2000-<3000	1 (5.6)		3 (21.4)				
	3000 -<4000	5 (27.8)		0 (0.0)				
	4000-<5000	3 (16.7)		2 (14.3)				
	5000-<6000	0 (0.0)		1 (7.1)				
	6000-<7000	1 (5.6)		1 (7.1)				
	7000-<8000	1 (5.6)		1 (7.1)				
	8000-<9000	1 (5.6)		0 (0.0)				
	9000-<10000	3 (16.7)		1 (7.1)				
	≥10000	1 (5.6)		0 (0.0)				
	Unknown	2 (11.1)		5 (35.7)				
Daily step goal (steps/day), median (IQR)			7000 (5000-8000)		6000 (5000-7000)		.64	

^a^HbA_1c_: glycated hemoglobin A_1c_.

^b^The *P* value of .73 is based on an analysis that included both participants with prediabetes and those with early-stage type 2 diabetes mellitus. Therefore, the nonsignificant group difference (*P*=.73) applies to the combined group of prediabetes and early-stage type 2 diabetes mellitus, not to each condition separately.

^c^T2DM: type 2 diabetes mellitus.

^d^Not applicable.

Figure 3 shows a comparison of the daily step achievement rates between the digital peer-supported and control groups. The median daily step achievement rate was significantly higher in the digital peer-supported app group (57.2%, IQR 32.2%-90%) than in the control group (26.7%, IQR 10%-64.4%; *P*=.04).

**Figure 4:**
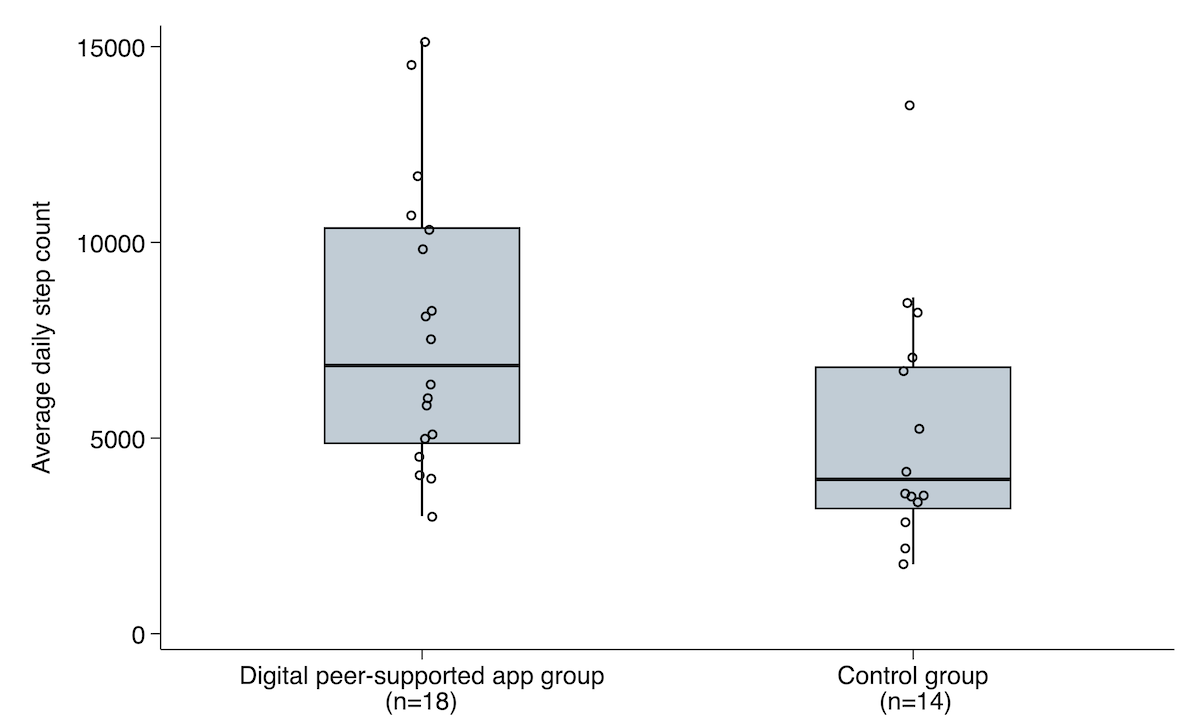
Comparison of average daily step counts between the digital peer-supported app and control groups. The median of the mean daily step count was significantly higher in the digital peer-supported app group (6854, IQR 4846-10388 steps) than in the control group (3946, IQR 3176-6832 steps), as determined by the Mann-Whitney U test (*P*=.03).

**Figure 5:**
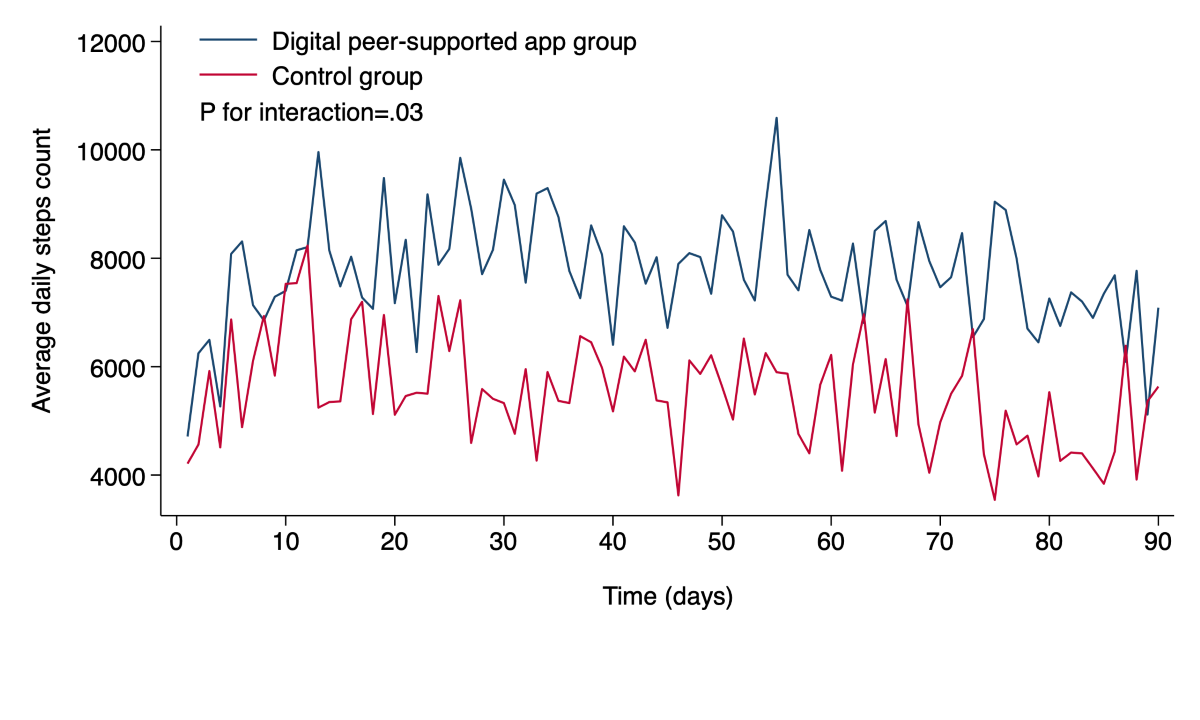
Group-level trends in average daily step counts over time. Each line represents the mean daily step count per day within each group.

Figure 4 compares the average daily step counts between the digital peer-supported and control groups. The median daily step count was significantly higher in the digital peer-supported app group (6854, IQR 4846-10388 steps) than in the control (3946, IQR 3176-6832 steps; *P*=.03). A linear mixed-effects model was constructed to further evaluate step count trajectories over time (Figure 5). The group-by-time interaction was statistically significant (*P* for interaction=.03), indicating a differential trend in the step counts between groups. In the digital peer-supported app group, no significant linear trend was observed in the daily step counts across the study period (*P* for trend=.26). In contrast, the control group showed a significantly decreasing trend over time (*P* for trend<.001).

Table 2 summarizes changes in secondary health indicators and lifestyle habits in the digital peer-supported app and control groups. Although no significant between-group differences were observed for HbA_1c_ levels, blood pressure, or lifestyle habits, pre-post changes in BMI showed a trend toward improvement in the digital peer-supported app group (*P*=.07). The digital peer-supported app group showed minor improvements in perceived health status, daily staple food consumption, and exercise frequency, whereas the control group experienced slight declines in perceived health and exercise frequency. Confidence in exercising increased in the digital peer-supported app group, but slightly decreased in the control group. Sleep duration of ≥6 hours increased slightly in the digital peer-supported app group but remained unchanged in the control group. Daily alcohol consumption decreased slightly in the digital peer-supported app group, but increased in the control group.

**Table 2:** Changes in anthropometric, metabolic, and lifestyle-related outcomes between the digital peer-supported app group and control group.

Variable	Digital peer-supported app group				Control group				*P*_Between-group_
	Baseline	Follow-up	*P*_Within-group_	Baseline		Follow-up	*P*_Within-group_		
							
BMI (kg/m²)^a^, median (IQR)	24.4 (22.0-26.1)	24.1 (21.7-25.9)	.07	23.4 (21.8-27.0)		23.0 (21.8-26.7)	.12	.92	
HbA_1c_^b^, median (IQR)	6.1 (5.7-6.4)	6.0 (5.6-6.3)	.81	6.2 (5.7-6.6)		6.2 (5.8-6.4)	.71	.73	
Systolic blood pressure (mm Hg)^c^, median (IQR)	120 (110-128)	127 (120-134)	.45	126 (121-134)		124 (113-136)	.35	.32	
Diastolic blood pressure (mm Hg)^c^, median (IQR)	77 (68-81)	75.5 (68-84)	.75	76 (72-79)		75 (73-78)	.86	.80	
Perceived good overall health status, n (%)^d^	9 (50)	10 (55.6)	.65	6 (42.9)		4 (28.6)	.22	.10	
Every day of balanced meal consumption, n (%)^d^	9 (50)	10 (55.6)	.65	5 (35.7)		5 (35.7)	.22	.24	
Always careful about food and healthy foods, n (%)^d^	3 (16.7)	5 (27.8)	.29	3 (21.4)		2 (14.3)	.11	.95	
Physical activity at least twice a week, n (%)^d^	8 (44.4)	12 (66.7)	.33	6 (42.9)		5 (35.7)	.57	.42	
Very confident in exercising, n (%)^d^	3 (16.7)	6 (33.3)	.34	4 (28.6)		3 (21.4)	.51	.17	
Average sleep duration ≥6 hours, n (%)^d^	8 (44.4)	10 (55.6)	.26	6 (42.9)		6 (42.9)	.95	.22	
Drink alcohol daily, n (%)^d^	5 (27.8)	3 (16.7)	.41	2 (14.3)		3 (21.4)	.67	.36	
Perceived as very stressful in daily life, n (%)^d^	3 (16.7)	3 (16.7)	.51	1 (7.1)		2 (14.3)	.39	.25	

^a^BMI (digital peer-supported app group: n=16 and control group: n=11).

^b^Glycosylated hemoglobin A_1c_ levels (digital peer-supported app group: n=12 and control group: n=10).

^c^Systolic and diastolic blood pressure (digital peer-supported app group: n=14 and control group: n=11).

^d^Questionnaire data for health status (digital peer-supported app group: n=18 and control group: n=14).

## Discussion

### Principal Findings

This study showed that users of a digital peer-supported app had significantly higher daily step goal achievements and average daily step counts compared to controls who did not receive digital peer support among individuals with prediabetes and early-stage T2DM, providing real-world evidence of the efficacy of this application. Given the limited research on individuals with prediabetes and early-stage T2DM, our findings expand the available evidence, suggesting that a digital peer-supported app may promote light physical activity, such as walking, and support its maintenance.

Our findings in individuals with prediabetes and early-stage T2DM are consistent with those of previous studies on patients with T2DM, which reported that eHealth and mobile health interventions can sustain and increase physical activity [19-24]. For example, a previous study reported that an interactive smartphone game aimed at increasing daily steps in people with T2DM resulted in a significant increase in activity levels compared to a control group that received standard lifestyle counseling [22]. In addition, another study showed that compared with the control group, the intervention group increased their daily step counts by using an app designed to increase physical activity [23].

Several features of digital peer-supported apps may explain why participants in the digital peer-supported app group were more likely to achieve their daily step goals and maintain a higher average step count than those in the control group without the app. First, real-time feedback and peer approval (similar to receiving a “Like” on Facebook) likely played an important role. Participants could visually monitor their progress, share daily activities, and receive feedback and approval through chats from team members, which may have maintained motivation by facilitating comparison, mutual support [25,26], and a sense of accomplishment while encouraging walking behavior [27]. In addition, notifications and reminders likely helped maintain participants’ motivation to engage in physical activity [25,27].

In the digital peer-supported app group, pre-post analysis showed no significant changes in HbA_1c_ or blood pressure; however, a trend toward BMI reduction was observed. A meta-analysis suggested that walking significantly decreases HbA_1c_ levels, BMI, and diastolic blood pressure in patients with T2DM [7]. The BMI reduction observed in our study may be due to the sustained increase in the daily step count over the 3 months. Finally, the absence of a significant HbA_1c_ alteration was consistent with the short study duration (3 months) and the early stage of T2DM in our cohort. As HbA_1c_ is a lagging, composite index averaging glycemia over 8-12 weeks, a single value has limited sensitivity to early metabolic dysfunction [28], thereby potentially masking subtle activity-related improvements. Future studies should incorporate more sensitive, high-frequency glycemic endpoints (eg, continuous glucose monitoring–derived time-in-range and glycemic variability) [17,29] to better detect the glycemic effects of app-induced physical activity increase.

Future studies are required to determine whether short-term or sustained use of a digital peer-supported app is more effective in supporting lasting behavioral changes and health. A digital peer-supported app may act as a short-term catalyst that initiates behavioral change or serves as a long-term support system that reinforces and sustains it. In this study, the digital peer-supported app may have triggered behavioral changes during the intervention period, potentially facilitating the development of long-lasting habits. However, these habits may not persist without continuous digital peer support.

### Limitations

This study examined the potential role of digital peer-supported apps in increasing and maintaining daily step counts. However, some challenges remain unaddressed. First, methodological limitations, including a small sample size, low statistical power, and potential confounding factors such as academic background, digital literacy, and motivation, cannot be ruled out. In addition, group enrollment was nonrandomized, and participation was voluntary. A formal sample size calculation was not conducted because of a lack of previous studies on the effectiveness of digital peer support on health outcomes. An RCT with a larger sample size and a parallel control group is ongoing to address these limitations (UMIN000046892) and to improve the validity and generalizability of the results. Second, the primary outcome (individual step count goals) was determined by the participants. Accordingly, the appropriateness and validity of these goals were not objectively verified. Third, we did not collect data on medication use or adjustments. Therefore, glucose-lowering or antihypertensive therapy initiation or titration during the follow-up might have contributed to the changes observed in HbA_1c_ and blood pressure, along with other unmeasured factors. Fourth, the daily step counts recorded by smartphone applications might have been underestimated due to variations in phone placement on the body or intermittent activity tracking. Finally, generalizability remains limited. Although most control participants used a smartphone pedometer app (ie, 4 feature-phone users received stand-alone pedometers), the intervention required a smartphone and a higher level of digital literacy to engage with the social features of the app.

### Conclusions

This study provides real-world evidence demonstrating that a digital peer-supported app may improve daily step goal achievement and average daily step counts in individuals with prediabetes and early-stage T2DM. The digital peer-supported app offers real-time feedback, peer approval, and continuous engagement to support sustained participation in light physical activities, such as walking. Given the limited number of studies on this population, our findings add to the evidence on the potential of digital peer support to promote and sustain light physical activity. Further studies are required to evaluate whether short-term app use is sufficient to initiate lasting behavioral changes or if long-term engagement is necessary to sustain health benefits.

## Appendix

Multimedia Appendix 1 Examples of user interfaces of digital peer support applications.

## Abbreviations

HbA1c: glycosylated hemoglobin A1c
RCT: randomized controlled trial
T2DM: type 2 diabetes mellitus
UMIN: University Hospital Medical Information Network
