# Supplementary material for: The Impact of a Digital Peer-Supported App on Daily Steps and Lifestyle Changes Among Individuals With Prediabetes and Early-Stage Type 2 Diabetes: Prospective, Nonrandomized Controlled Trial
Source: JMIR Form Res. 2025 Dec 15;9:e75953. doi: 10.2196/75953 (PMC12750071; doi:10.2196/75953)
Supplement: Multimedia Appendix 1 [file formative_v9i1e75953_app1.pdf]

"Minchalle" App

1. **Posting to Chat has Four Functions**
   - Challenge Photo
   - Normal Photo
   - Comment
   - Stamp

2. **Exchange for Coins**
   - Function for Donation

3. **Function for Recording**
   - Records List
   - My Data
   - Calendar

4. **Assistant Robot**

5. **Team owner function**

1. Posting to Chat has Four Functions

・Challenge Photo

"Challenge photos" can be posted once a day and must be taken on the same day, with a limit of one submission per day. You will earn coins when your team members approve your challenge photos. To post a comment, follow these steps:

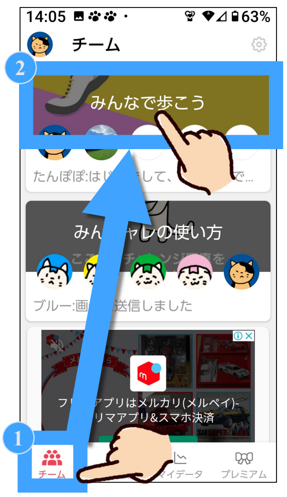

(A)

1. Touch "Teams" in the lower left corner
2. Touch on the name of the participating team

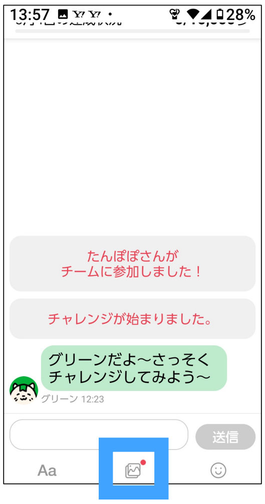

(B)

Touch the posted photo button at the bottom center

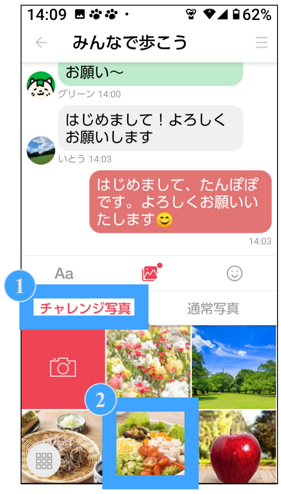

(C)

1. Touch ”challenge photo”
2. Select the photo you like to post

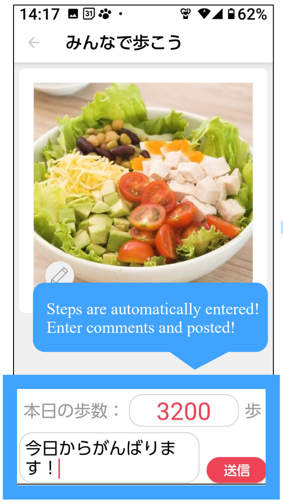

(D)

Enter comments and touch "Post"

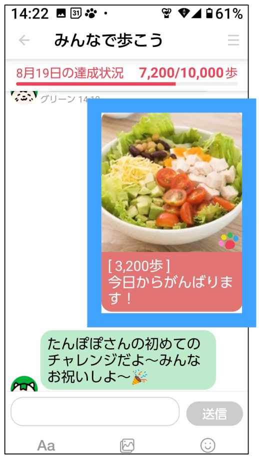

(E)

The number of steps taken at the time the challenge photo is posted will be recorded

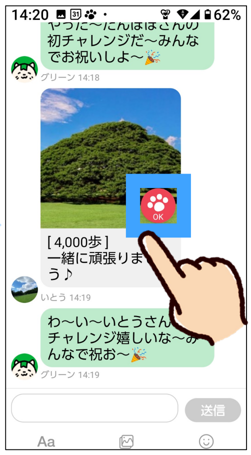

(F)

Approve photos posted by team members

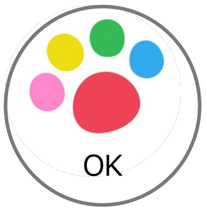

When approved, the paw pads turn colorful.

・**Normal Photo**

"Normal photos" can be posted as often as you like, and these can include images from the past. To post a comment, follow these steps:

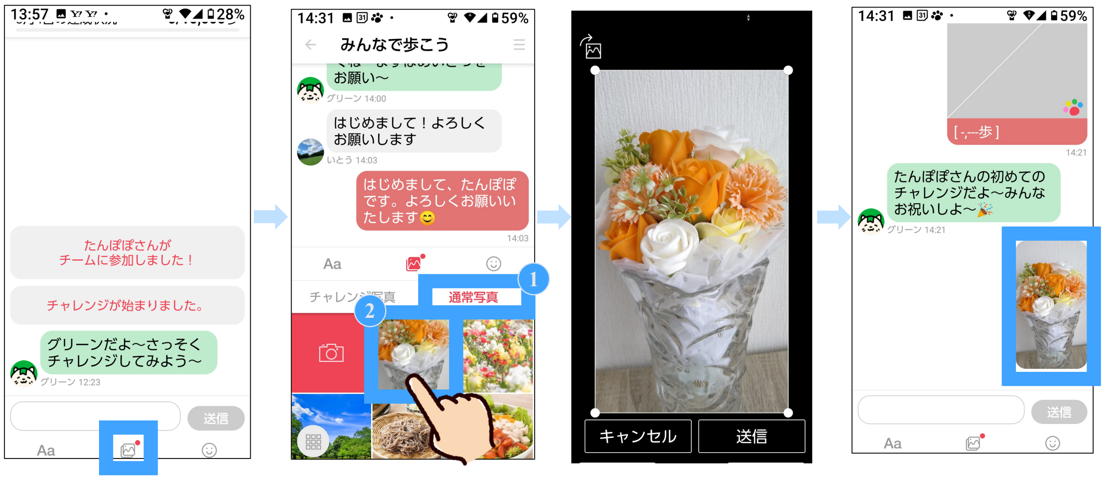

(A)

Touch the posted photo button at the bottom center

(B)

1. Touch "Normal Photo"
2. Select the photo you like to post

(C)

A "normal photo" will be posted

＊ Difference between Challenge Photo and Normal Photo

	Challenge Photo	Normal Photo
Number of posts	Limited to once a day	Unlimited
Useable photos	Only photos taken on the same day	Past photos are acceptable
Display of the number of steps*	○	×
Approval button	○	×
Comment	○	×

*Note： This applies only if you are part of a team that tracks your steps.

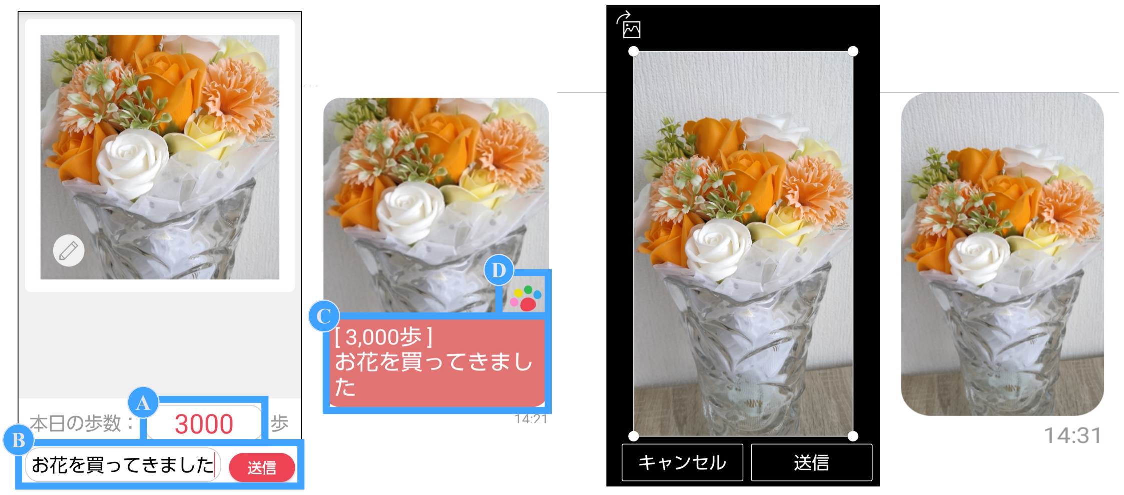

Challenge **Normal photo**

Note：ⒶEnter steps;ⒷEnter comments;ⒸSteps and remarks after posting;ⒹApproval button.

・Comment

Users can leave comments on the chat screen. Your comments will appear highlighted in red, whereas comments from other members will be in white. To post a comment, follow these steps:

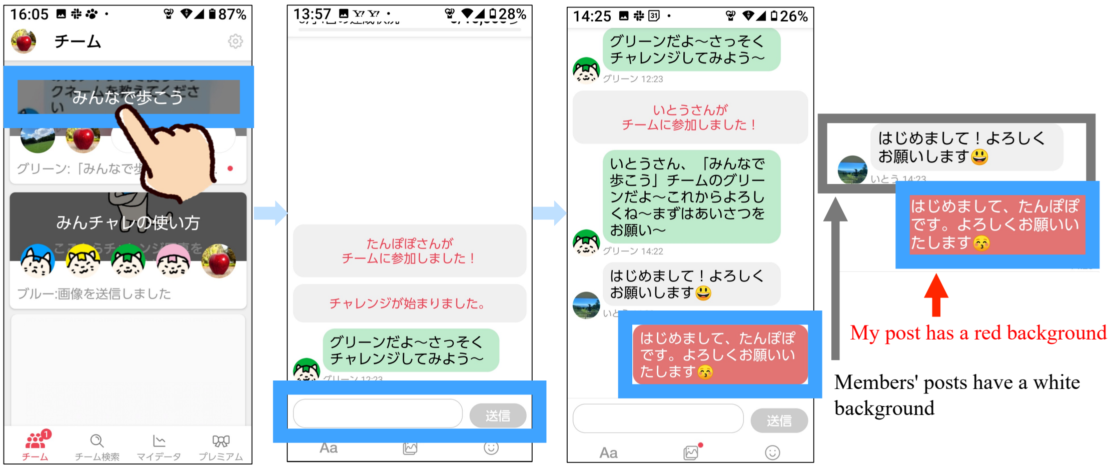

(A)

Touch on the name of the participating team

(B)

Enter comments and touch "Post"

(C)

A "Comments" will be posted

・Stamp

Users can post stamps on the chat screen.

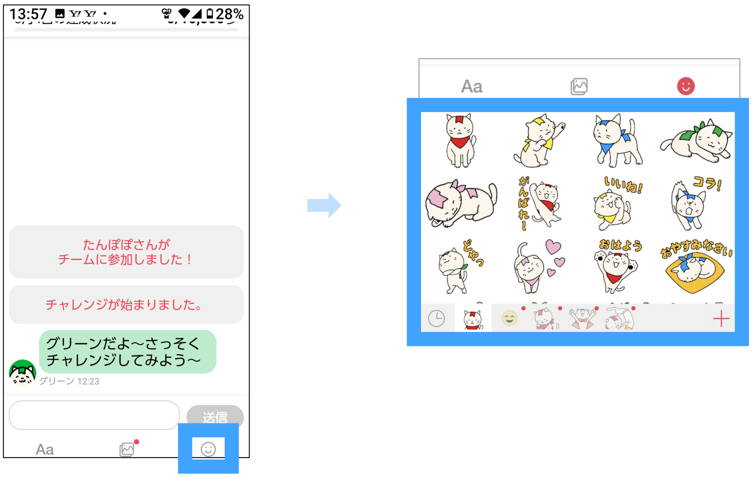

(A)

Touch on the stamp button located at the lower right corner of the chat screen

(B)

Select the stamp that you like to use for posting

2. Exchanged for Coins

The app enables users to accumulate coins that can be redeemed for in-app wallpaper or stamps. Additionally, users have the option to utilize the app's donation function.

・Donation function

Coins can be donated to local governments and various organizations for their donation projects.

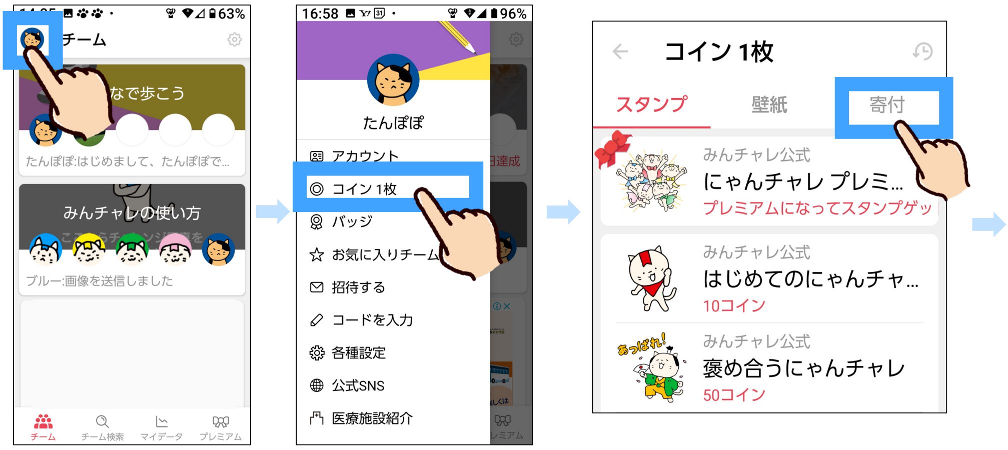

(A) Touch on the profile picture in the top left corner

(B) Touch "◎ coins"

(C) Touch "Donate"

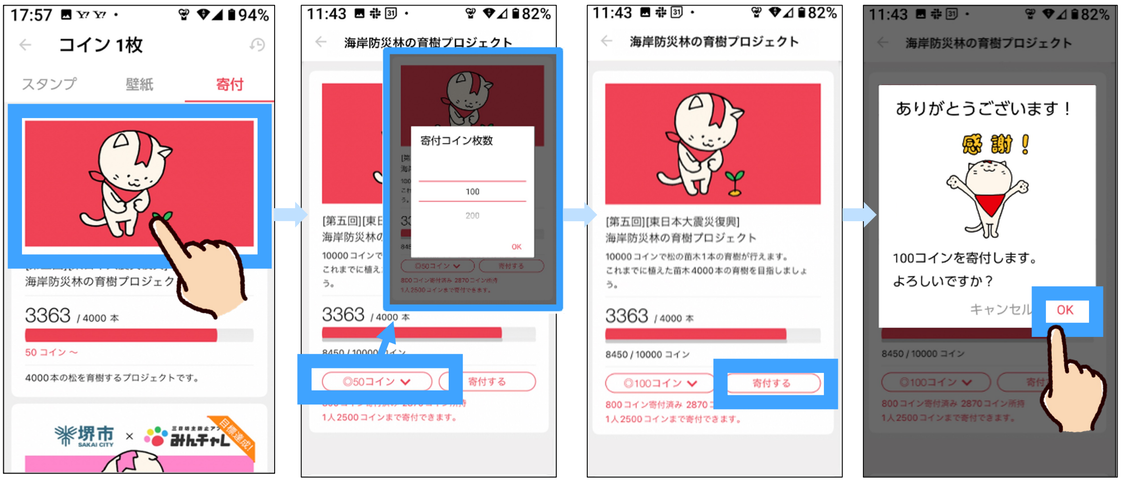

(D) Select and touch the donation of your choice

(E) Choose the number of coins you like to donate

(F) Touch the "Donate" button

(G) Touch the "OK" button again to complete donating

3. Function for Recording

The user's records can be traced back to the following three key points:

・**List of Past Records**

Track a record of the number of steps you have contributed to the past as well as the total number of steps taken by all participating teams.

・**My Data**

Users can evaluate their performance in achieving their set goals.

・**Calendar**

Users can access a calendar to review their past challenge photo.

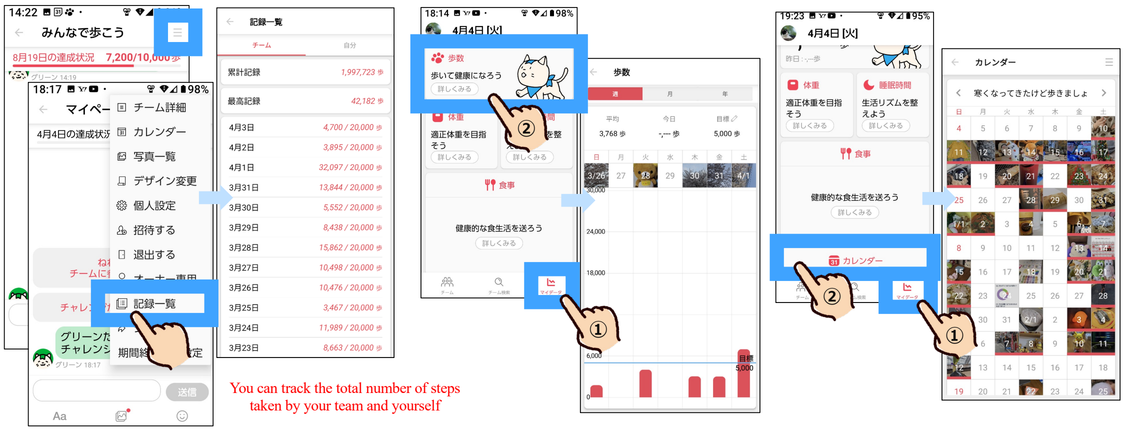

(A) List of Past Records

Touch the three lines located at the top right corner of the chat screen and then select either your team or yourself

(B) My Data

Touch on "My Data" at the bottom right corner. Then, select "Steps"

(C) Calendar

Touch on "My Data" at the bottom right corner. Then, select "Calendar"

4. **Assistant Robot**

The team has access to an assistant robot that provides encouragement and feedback on their daily tasks. Team members can select and change their assistant robot as needed. However, the robot is not equipped to respond to user inquiries. These assistant robots are designed with distinct personalities, each tailored to enhance the team's experience. Here are the details of the team's robot's personality:

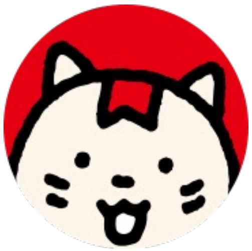

(A)

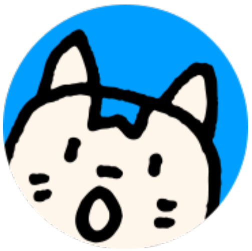

(B)

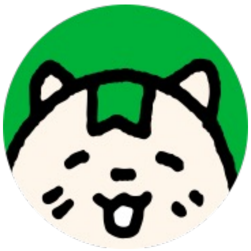

(C)

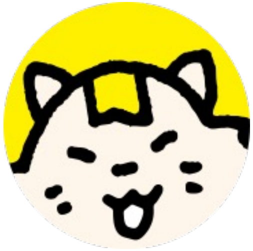

(D)

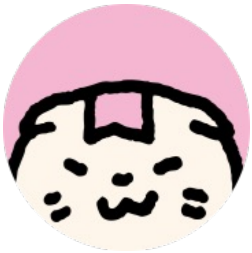

(E)

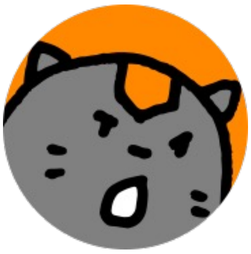

(F)

(A) Red assistant robots are designed to motivate and inspire users, developing good habits.
(B) Blue assistant robots focus on helping users create and maintain daily habits effectively.
(C) Green assistant robots support users in developing desired habits at their own pace.
(D) Yellow assistant robots aim to make the process of developing desirable habits enjoyable.
(E) Pink assistant robots are gentle and supportive, assisting users in creating desired habits.
(F) Black assistant robots have a more challenging approach and interfere with their activities.

5. Team owner function

Note that only the owner of the team has the authority to set or modify the following four points:

・Team Name

When forming a team of up to five members, the team can select a name. However, only the team owner has the authority to change the team's name within the application.

・Team Goals

When forming a team with up to five members, the owner can set a team goal, such as achieving 10,000 steps per day. However, only the owner can modify the team's goal within the application.

・Assistant Robot

When creating a team of up to five members, the owner can select an assistant robot, such as a green assistant robot. However, only the owner has the authority to change the team's assistant robot within the application.

・Automatic exit function

To ensure active participation, each team should have the capability to automatically remove members who have not made a post within a specified time frame. Teams can select this time frame from the available options: "2 days," "4 days," "8 days," or "15 days."

The actual operating procedure as follows:

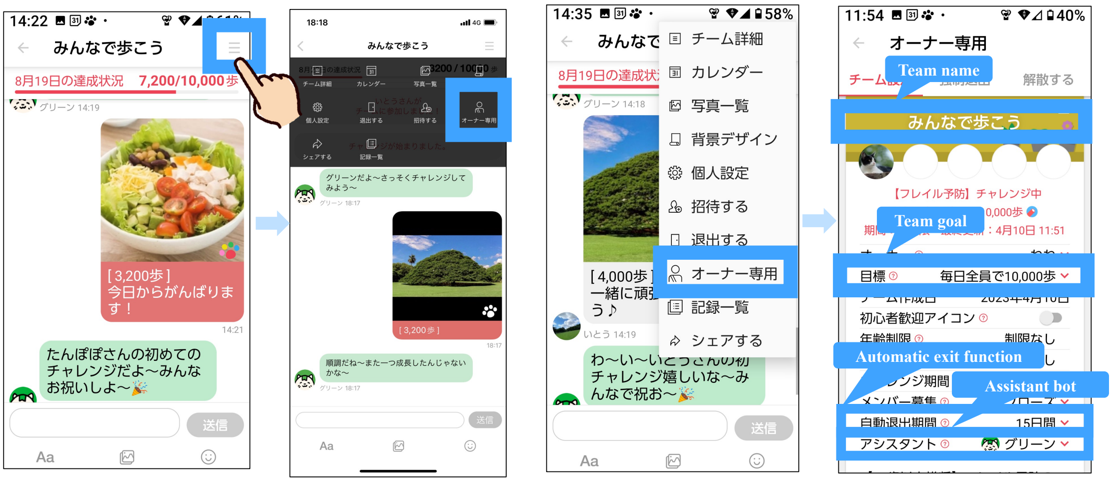

(A) Touch the three lines in the upper right corner

(B) iPhone Version Touch "Owner Only"

(C) Android Version Touch "Owner Only"

(D) Four change and setting buttons
